# 经左胸全隆凸切除、右主支气管切除重建术1例报道

**DOI:** 10.3779/j.issn.1009-3419.2010.03.14

**Published:** 2010-03-20

**Authors:** 向宁 付, 波 艾, 霓 张, 威 孙

**Affiliations:** 430030 武汉，华中科技大学同济医学院附属同济医院普胸外科 Department of Thoracic Surgery, Tongji Hospital, Tongji Medical College, Huazhong University of Science and Technology, Wuhan 430030, China

经左胸全隆凸切除重建术临床少见^[1]^，同期行右主支气管切除、右上叶-中间段支气管重建右主支气管、再与气管下段吻合重建隆凸术则国内外未见报道。华中科技大学同济医学院附属同济医院普胸外科在2009年2月成功施行该手术1例，现报道如下。

## 1 病例资料

患者，男，47岁，因“刺激性干咳2年余，高热2天”入院。查体生命体征稳定，气管左移，左肺呼吸音低，右肺呼吸音正常，双肺未闻及啰音。胸部X片及CT示气管下段腔内结节影并左全肺不张，未见左主支气管开口，纵隔明显左移（图 1、图 2A、图 2B）。纤维支气管镜示气管下段新生物，管腔阻塞严重（图 3）。胸部CT三维重建示气管下段软组织影，紧贴气管左壁（图 2C）。术前诊断：左主支气管肿瘤累及气管下段，左全肺不张。患者于2009年2月5日在全麻体外循环下左进胸行剖胸探查术。术中见左全肺不张，肿瘤位于左主支气管内，大小约6 cm×3 cm×3 cm，左主支气管完全堵塞，隆凸及右主支气管受累（图 4），遂行左全肺切除+隆凸-右主支气管切除重建术。游离左肺门后处理左肺动、静脉，游离左主支气管、隆凸、右主支气管及气管下段，于隆凸上2 cm处离断气管，分别于右上叶支气管根部、右中间段支气管根部离断右上叶支气管及中间段支气管，重建过程先将右中间段支气管与右上叶支气管侧-侧吻合重建上叶-中间段间嵴，形成新的右主支气管开口，再与气管下段端端吻合重建气道完整性，最后游离带蒂大网膜包埋吻合口。术后呼吸机辅助呼吸，24 h后顺利脱机。患者术后咳痰欠佳，术后第3天出现呼吸困难，予纤支镜吸痰，症状明显缓解。术后第4天行气管切开，每日辅助纤支镜吸痰，病情逐渐康复，术后30天顺利出院。手术标本病检示（左主支气管内）腺样囊性癌（圆柱瘤），累及右主支气管，各残端阴性。

**1:**
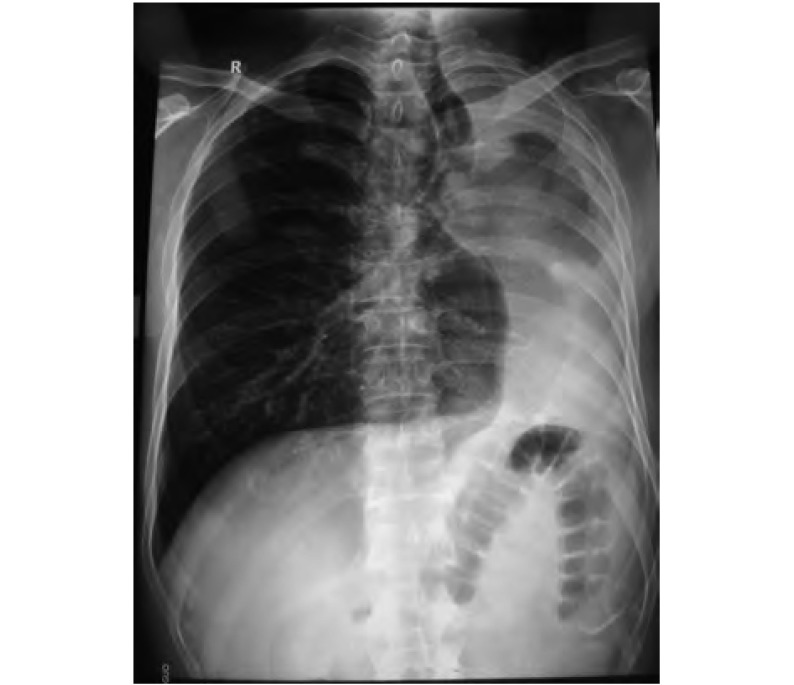
术前胸片示气管腔内见肿块影，左主支气管未见，左肺不张，右肺过度扩张 X-ray before operation showed mass in trachea, the left bronchea wasn't showed, left lung collapsed, and right lung over expanded

**2:**
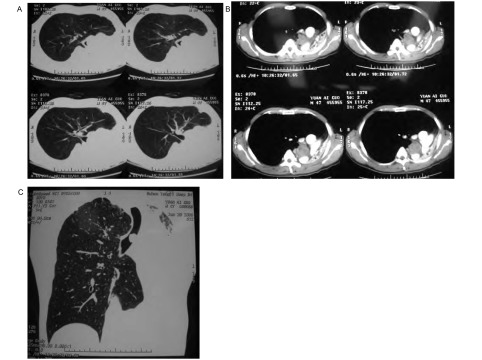
术前CT检查示气管近隆突处见肿块影，左主支气管开口无显示，左全肺不张，右肺过度扩张 CT scan before operation showed mass in trachea near carnia, the openning of left bronchea wasn't showed, left lung collapsed, and right lung over expanded

**3:**
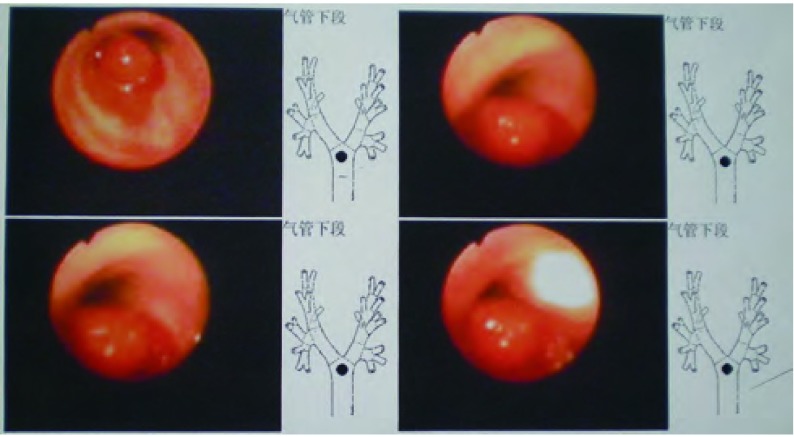
术前纤支镜检查示气管下段见新生物突入管腔，管腔几乎完全闭塞 Bronchoscopy before operation showed neoplasm in the lower trachea, the cavity was almost occlused completely

**4:**
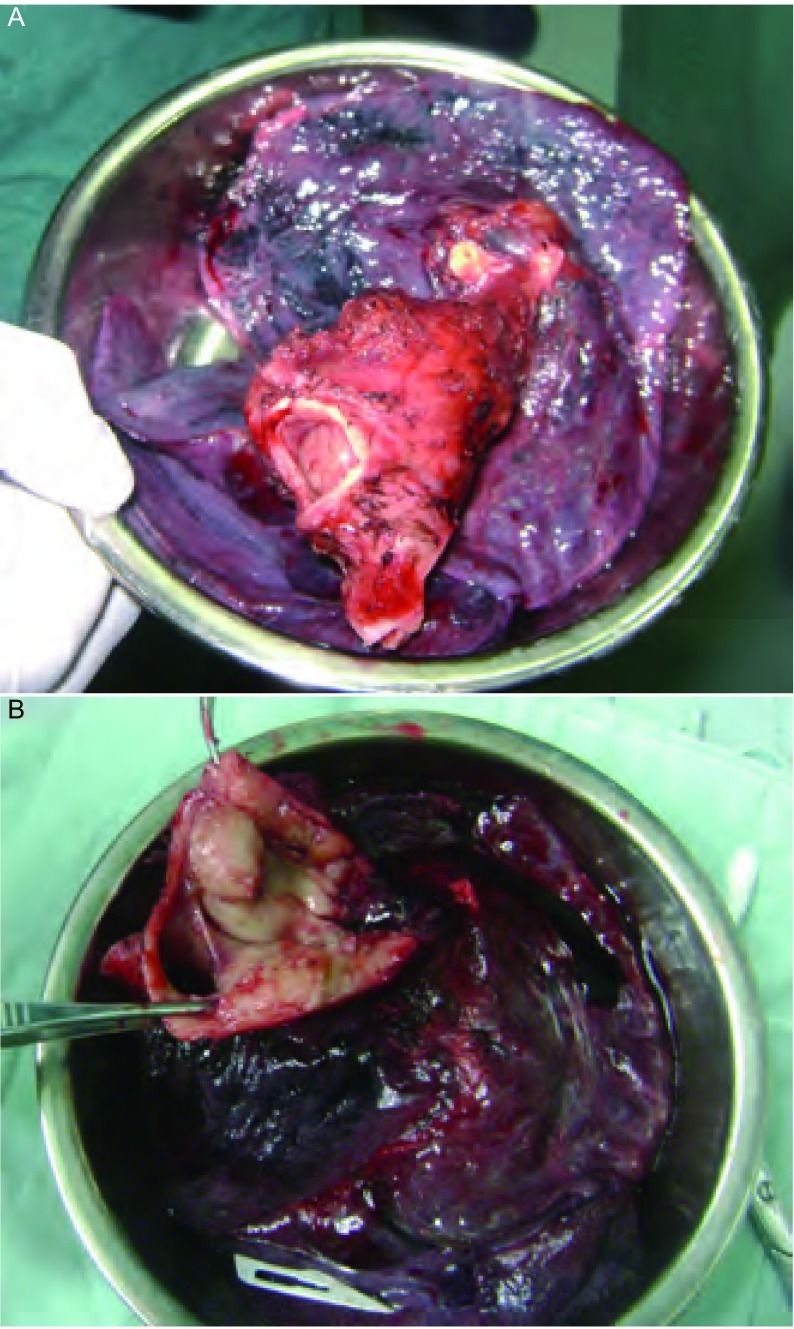
术中标本：肿瘤位于左主支气管，累及隆突及右主支气管 Sample in operation showed tumor located in left bronchea, carnia and right bronchea were involved

## 2 讨论

### 2.1 术式选择

隆凸切除重建术是胸外科的热点和难点^[2]^。一般行右胸后外侧切口，因气管、隆凸等显露较左进胸好^[3]^。经左胸行全隆凸、右主支气管切除，右上叶-中间段支气管-气管下段吻合重建隆凸术临床罕见。本病例同时存在左全肺不张和隆凸、右主支气管受累，需同期行左全肺切除，只能经左侧进胸。虽可辅助股动脉-股静脉转流部分体外循环技术（Cardiopulmonary Bypass, CPB）^[4, 5]^，但因其增加术后并发症，故仅作为术中备用。

### 2.2 术中要点

包括以下6点：①因左肺门空间有限，显露不良，故游离部分主动脉弓，并离断第1、2对肋间动脉，以改善手术野的显露；②胸导管与主动脉弓紧邻，为预防术后乳糜胸的发生，术中常规结扎胸导管^[6]^；③离断左肺动、静脉后，不急于离断左主支气管，以此作牵引，完成全隆凸及右主支气管的显露；④术中间断右中间段支气管内通气给氧，利用氧储备，完成“上叶-中间段间嵴”及隆凸的重建，重建过程中防止渗血灌入右肺；⑤吻合“新的右主支气管开口”与下段气管时，进针不宜过深，以避免伤及后方的奇静脉，引起出血、窒息；⑥左进胸经膈肌游离带蒂大网膜方便，而大网膜是气管吻合口包埋的良好材料，转移长度长，生理强度高，愈合粘连快，包埋充分，并且可促进吻合口血管再生^[7]^，利于愈合，有效预防吻合口瘘的发生。

本病例痊愈出院，随访4月余疗效满意，经左胸行左全肺袖式切除或加右主支气管切除重建术对部分特定病例可以尝试。

## References

[b1] 1Gu KS ed. KS Koo Thoraco-cardiac Operative Surgery. 1st ed. Shanghai: Shanghai Scientific and Technological Publicating House, 2003. 648-649.顾恺时主编. 顾恺时胸心外科手术学. 第1版. 上海: 上海科学技术出版社, 2003. 648-649.

[2] Jia H, Xu L, Qiu NL (2008). The role of carinal reconstruction in the treatment of lung cancer. J Basic and Clin Oncol.

[3] Yamamoto K, Kosaba S, Ikeda T (2001). Evaluation of therapeutic method for malignant tumors involving the tracheal carina. Kyobu Geka.

[4] Minamiya Y, Saito H, Ogawa J (2008). Experience of twenty-one cases of tracheobronchoplasty. Kyobu Geka.

[5] Du ZZ, Ren H, Song JF (2006). Perioperative management for primary tracheal malignant tumors resected under cardiopulmonary bypass. Chin J Oncol.

[6] Zhao B, Sun W, Fu XN (2006). Effect of preventive management during operation for post-operative chylothorax in patients with lung cancer of state Ⅲ. Chin J Postgraduates Med.

[7] Xu L, Yu MF, Yuan FL (2006). Experience of carinal resection and reconstruction in the treatment of carinal tumor and bronchogenic carcinoma. Chin J Lung Cancer.

